# Implementation of a mobile 0.15-T intraoperative MR system in pediatric neuro-oncological surgery: feasibility and correlation with early postoperative high-field strength MRI

**DOI:** 10.1007/s00381-012-1815-8

**Published:** 2012-06-10

**Authors:** P. L. Kubben, H. van Santbrink, M. ter Laak-Poort, J. W. Weber, J. S. H. Vles, B. Granzen, J. J. van Overbeeke, E. M. J. Cornips

**Affiliations:** 1 Department of Neurosurgery, Maastricht University Medical Center, PO Box 5800, 6202 AZ Maastricht, the Netherlands; 2 Department of Child Neurology, Maastricht University Medical Center, PO Box 5800, 6202 AZ Maastricht, the Netherlands; 3 Department of Pediatrics, Maastricht University Medical Center, PO Box 5800, 6202 AZ Maastricht, the Netherlands

**Keywords:** Intraoperative MRI, Postoperative MRI, Feasibility, Oncology, Pediatric neurosurgery

## Abstract

**Introduction:**

We analyze our preliminary experience using the PoleStar N20 mobile intraoperative MR (iMR) system as an adjunct for pediatric brain tumor resection.

**Methods:**

We analyzed 11 resections in nine children between 1 month and 17 years old. After resection, we acquired iMR scans to detect residual tumor and update neuronavigation. We compared final iMR interpretation by the neurosurgeon with early postoperative MR interpretation by a neuroradiologist.

**Results:**

Patient positioning was straightforward, and image quality (T1 7-min 4-mm sequences) sufficient in all cases. In five cases, contrast enhancement suspect for residual tumor was noted on initial postresection iMR images. In one case, a slight discrepancy with postoperative imaging after 3 months was no longer visible after 1 year. No serious perioperative adverse events related to the PoleStar N20 were encountered, except for transient shoulder pain in two.

**Conclusions:**

Using the PoleStar N20 iMR system is technically feasible and safe for both supra- and infratentorial tumor resections in children of all ages. Their small head and shoulders favor positioning in the magnet bore and allow the field of view to cover more than the area of primary interest, e.g., the ventricles in an infratentorial case. Standard surgical equipment may be used without significant limitations. In this series, the use of iMR leads to an increased extent of tumor resection in 45 % of cases. Correlation between iMR and early postoperative MR is excellent, provided image quality is optimal and interpretation is carefully done by someone sufficiently familiar with the system.

## Introduction

Pediatric intrinsic brain tumors differ from their adult counterparts in several ways that are of major therapeutic importance. The value of extensive tumor resection, which is still under debate for malignant intrinsic brain tumors in adults, has been confirmed for a variety of pediatric brain tumors. Moreover, the prognosis of histologically similar tumors is often more favorable in children than in adults [17]. Neurosurgical treatment consists of a maximal safe resection, balancing maximal resection with preservation of neurological function. In this regard, the underlying principle “primum non nocere” may be interpreted as “take out what you want to take out, and leave behind what you want to leave behind.” Image-guided surgery can certainly help to achieve this goal [2], with preoperative images used for surgical planning and navigation. Unfortunately, these images become progressively inaccurate during the course of surgery because of brain shift following loss of cerebrospinal fluid, resection of pathological tissue, and development of edema. This is exactly where intraoperative MRI (iMRI) may be of use: to demonstrate residual tumor and to update images for navigation [1, 3–8, 11, 13, 15, 20, 21, 24].

In contrast to the adult literature, even to date there have been few reports on the use of iMRI in pediatric neuro-oncological surgery [9, 10, 14, 18, 19]. We report our preliminary experience with the low-field strength PoleStar N20 mobile iMR system, focusing on the feasibility of this particular equipment, whether its use had direct intraoperative consequences and whether postresection low-field strength iMR images were in accordance with early postoperative high-field strength MR (epMR) images.

## Material and methods

### Patient population

Between 2005 and 2010, we performed 11 iMR-guided craniotomies for brain tumor resection in nine children between 1 month and 17 years old. All children were operated by a pediatric neurosurgeon (all but one by EC). Patient characteristics and tumor location are detailed in Table 1.

**Table 1:** Patient characteristics

Case #	Sex	Age (years)	Tumor location	Positioning
1	M	<1	Right parietal	Supine
1 (recurrence)	M	<1	Right parietal	Supine
2	F	17	Right frontal	Supine
3	M	6	Posterior fossa	Prone
4	F	11	Left frontal	Supine
5	M	13	Left parafalcine perirolandic	Supine
6	M	6	Posterior fossa	Prone
7	M	13	Right temporal	Supine
8	M	5	Posterior fossa	Prone
8 (residual)	M	6	Posterior fossa	Prone
9	F	12	Right postrolandic	Supine

*F* female, *M* male

### Intraoperative MRI setup

**Fig. 1:**
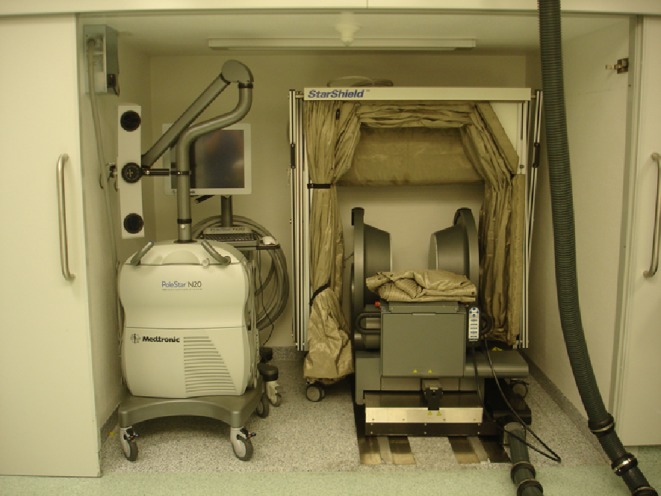
PoleStar N20 system and StarShield® radiofrequency shielding

**Fig. 2:**
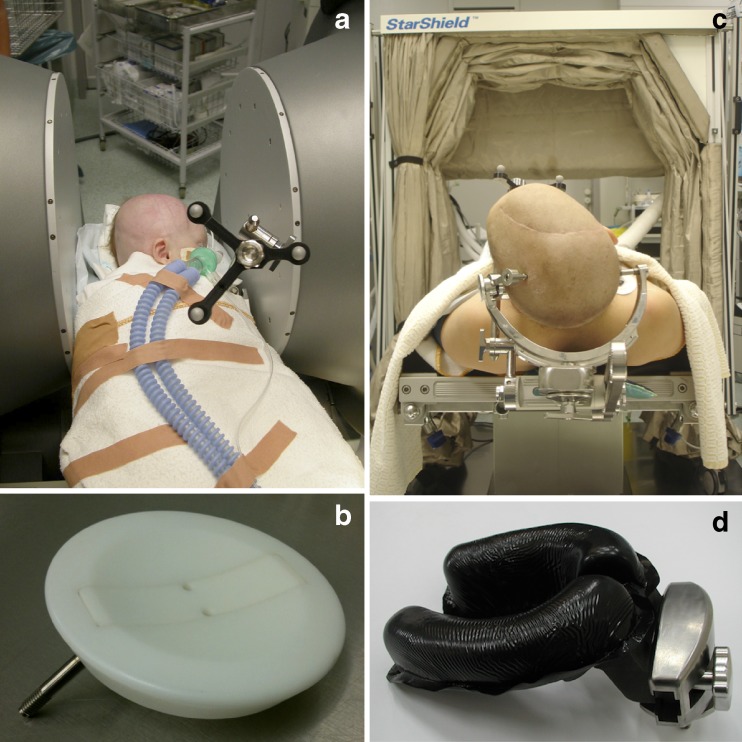
PoleStar N20 system details, including **a** aperture of the magnet bore, **b** concave plastic headrest, **c** headclamp with fixed diameter, and **d** padded pediatric horseshoe headrest

**Fig. 3:**
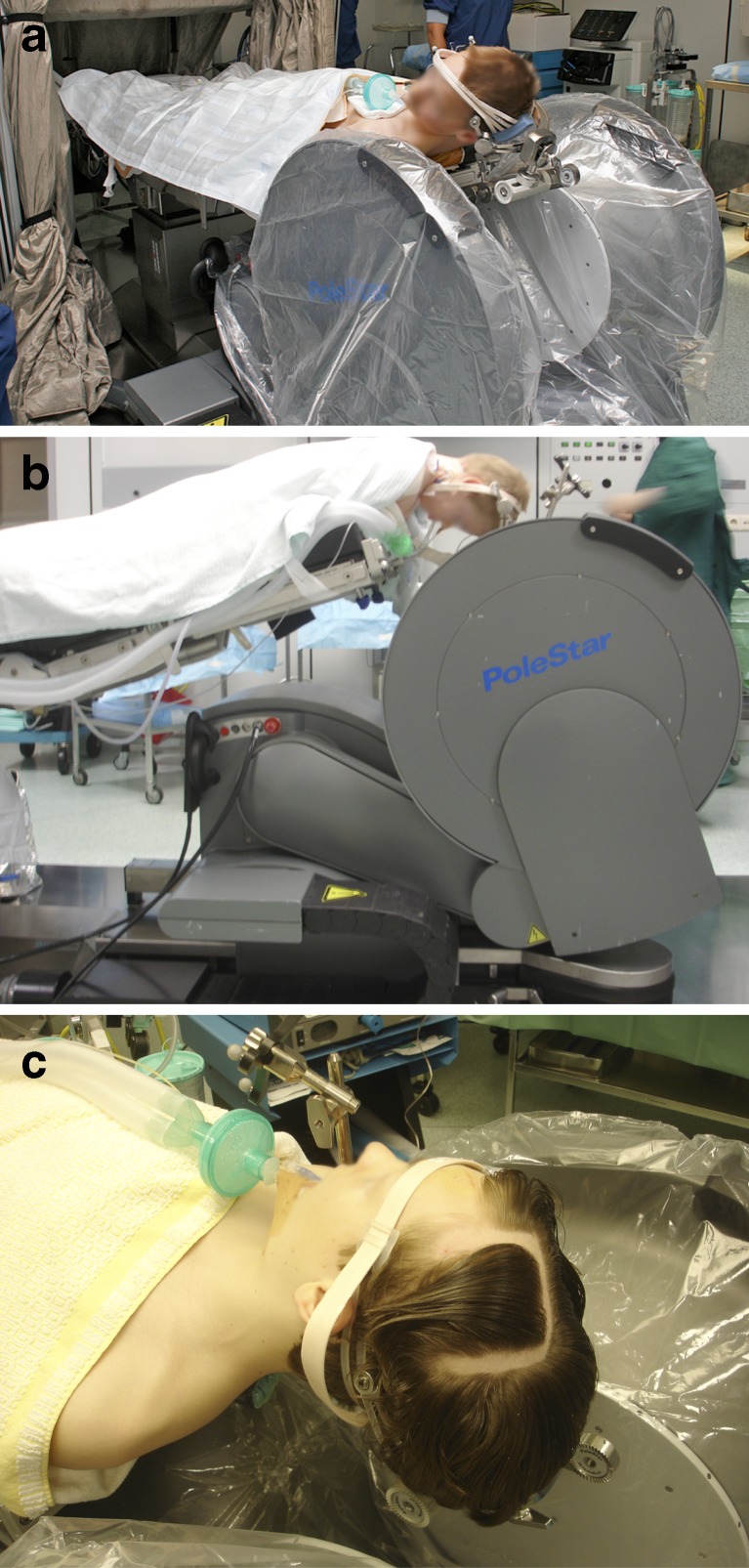
**a**–**c** Typical patient positioning, **a** supine, **b** prone, and **c** detail of a child’s head in the fixed diameter clamp inside the magnet bore

In 2005, a PoleStar N20 iMR system (Medtronic Navigation, Louisville, CO, USA) incorporating a mobile 0.15-T magnet and local radiofrequency shielding (so-called StarShield®) was installed in our center (Figs. 1 and 2). The system has an aperture of 25 cm between the magnet spools and a field of view (FOV) measuring 20 × 20 × 16 cm [16, 22]. The child’s head is fixated in an MR-compatible headclamp with fixed diameter (to fit in between the spools) and variable-length screws (Fig. 3c). For young children where the neurosurgeon decides not to use headclamping, a concave plastic headrest and more recently a padded pediatric horseshoe headrest are available. Because of the magnet’s low-field strength, regular equipment (including pneumatic drill, microscope, ultrasonic aspirator, and micro-instruments) can be used. On the other hand, an MR-compatible anesthesia monitor, cardiac electrodes, and thermometer are mandatory, and an armored tube (prone positioning) should be avoided because of interference during image acquisition.

### Intraoperative image acquisition and contrast administration

The PoleStar N20 can acquire different MR sequences: T1-weighted, T2-weighted, fluid attenuated inversion recovery, and so-called e-steady scans, which are very short sequences mainly used during patient positioning. Moreover, several scanning protocols are available to optimize scanning time, anatomical detail, and signal-to-noise ratio (SNR). In this series, we routinely used the so-called T1 7-min 4-mm sequence because it has the highest SNR available on the system and is well suited for most intra-axial pediatric brain tumors. We followed a strict protocol with regard to the timing of contrast administration. As there would be diagnostic MR scans with contrast available in all cases, we chose to obtain only non-contrast iMR images preresection (used for positioning, planning the craniotomy site, and comparison with postresection images), followed by non-contrast and finally contrast iMR images postresection. The latter images were obtained after the administration of gadopentetate dimeglumine (Magnevist, Bayer-Schering Pharma AG, Berlin, Germany) in a dose of 0.1 mmol/kg (“single-dose”) immediately before scanning. In order to obtain intraoperative scans, all instruments are removed from the surgical field, the magnet is raised to scanning position, the patient (with open skull) is covered with sterile drapes, and the StarShield® is closed.

Importantly, in case of an intended gross total resection, the surgeon (a dedicated pediatric neurosurgeon) did not stop initial resection until he or she believed to have taken out the tumor completely and was ready for closure. There were two reasons for this, a practical one and a theoretical one. The practical and for this preliminary study most important reason was not to interrupt surgical flow and to minimize blood loss. The theoretical reason was to allow a better assessment of the value of this iMR system in detecting unintended residual tumor.

### Postoperative MRI

As in routine pediatric neuro-oncological practice, we acquired postoperative MRI scans within 24 h on a 1.5-T MRI system (Intera release 11.1, Philips, Best, the Netherlands). For contrast-enhanced T1-weighted scans, we administered “single-dose” Magnevist immediately before scanning. All postoperative scans were independently reviewed by a neuroradiologist without seeing the intraoperative images, or knowing the neurosurgeons interpretation of these images (or any actions taken accordingly). As such, we compared iMR interpretation by the neurosurgeon with early postoperative MR interpretation by the neuroradiologist, both blinded for their respective images and image interpretation.

## Results

The results are summarized in Table 2.

**Table 2:** Schematic overview of 11 iMRI-guided craniotomies for brain tumor resection in nine children

Case #	Histology	iMRI	epMRI	Postoperative neurological deficit	Follow-up
1	MOA	GTR	GTR	No	Recurrence (next case)
1 (recurrence)	GNTOI^a^	Intended STR	STR	No	Alive (>5 years) progression free
2	GB recurrence	GTR^b^	GTR	No	11 months
3	PA	GTR^b^	GTR	No	Alive (>3 years)
4	MEP	GTR^b^	GTR	No	1 year
5	PA	GTR^b^	GTR	Transient dysphasia	Alive (>3 years)
6	Medullobl	GTR	GTR	No	Alive (>3 years)
7	GG grade 2	GTR	GTR	No	Alive (>2 years)
8	PA	Intended STR^b^	STR	Cerebellar mutism	Residual (next case)
8 (residual)	PA	GTR	GTR	No	Alive (>3 years)
9	DNET	GTR?	STR	No	Alive (>1 years) progression free

*DNET* dysembryoplastic neuroepithelial tumor, *GB* glioblastoma, *GG* ganglioglioma, *medullobl* medulloblastoma, *GNTOI* glioneuronal tumor of infancy, *GTR* gross total resection, *GTR?* probable GTR, *MEP* malignant ependymoma, *MOA* malignant oligoastrocytoma, *PA* pilocytic astrocytoma, *STR* subtotal resection

^a^Histological examination of the resected recurrence revealed a much more differentiated “glioneuronal tumor of infancy”

^b^Indicates that an increased EOTR was achieved after additional resection as suggested by iMRI

### Positioning, imaging, and FOV

Patient positioning was rather straightforward in all procedures (supra- and infratentorial) (Fig. 3a, b) as well as in all age categories (babies, toddlers, older children). Using the plastic headrest was convenient in case of babies and toddlers; however, in order to enable accurate navigation, we had to tape the head to the headrest without compromising the craniotomy site. The headclamp was convenient for older children; however, because of its non-adjustable diameter, we had to use longer screws to compensate for the relatively smaller size of the head as compared to adults (Fig. 3).

**Fig. 4:**
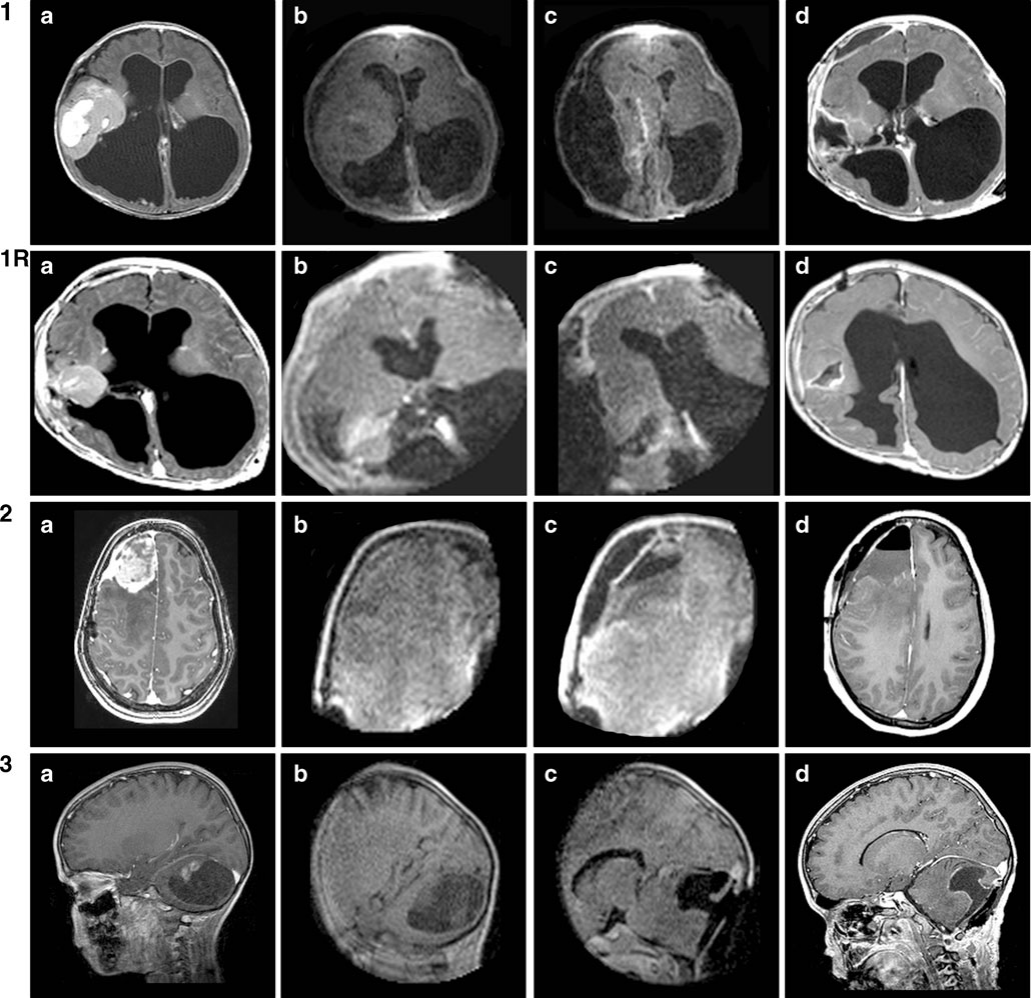

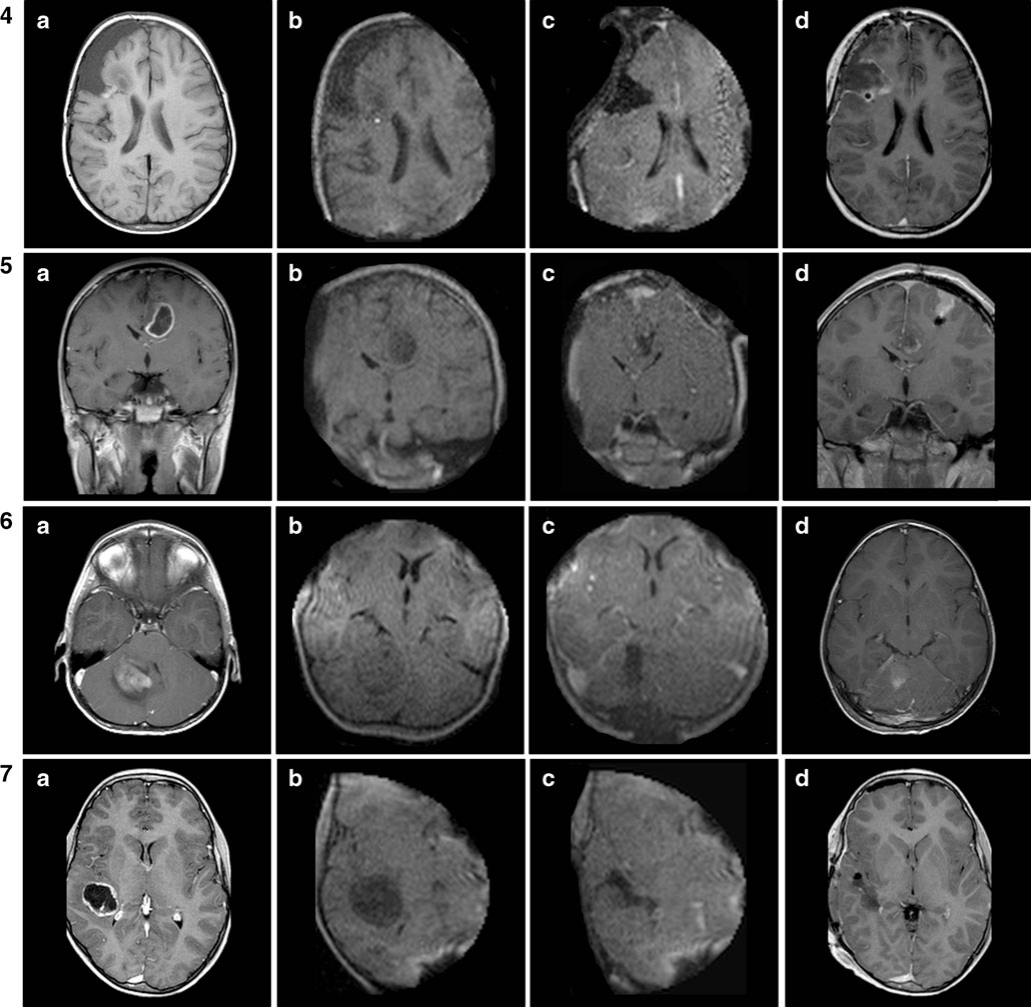

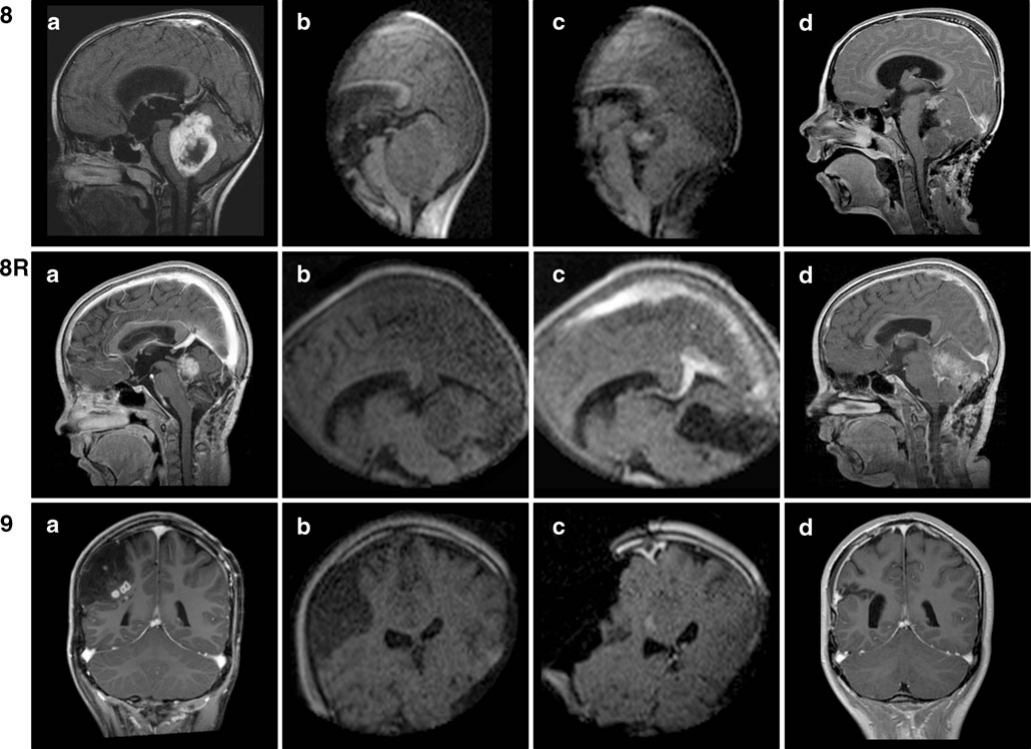
Overview of preoperative (**a**), intraoperative (**b**), postresection intraoperative (**c**), and early postoperative MR images (**d**) in all cases (*n* = 11). The intraoperative images are T1 7-min 4-mm sequences (preresection without contrast, postresection with contrast). The postoperative images are T1 sequences with contrast

According to the neurosurgeon’s subjective judgment image quality was sufficient in all cases provided care was taken to keep noise levels below 10 % “above typical.” Above that value, image quality rapidly deteriorates and interpretation becomes less reliable. Although any scan series (either axial, coronal, or sagittal) is readily reconstructed by the system’s software, providing accurate navigation in all three orthogonal planes, in our experience reconstructed image quality is insufficient to reliably assess residual tumor. Therefore, we routinely obtained images in two or even three orthogonal planes before and after resection, depending on the overall aspect and location of the tumor. Figure 4 shows some of the most illustrative images (low-field strength as well as high-field strength) for each respective case pre- and postresection. Of note, in all posterior fossa cases (*n* = 4), the FOV was large enough to visualize not only tumor and posterior fossa contents but also the supratentorial ventricles (example in Fig. 4c 8Rc).

### Intraoperative and postoperative MRI

Figure 4 offers an overview of preoperative, preresection intraoperative, postresection intraoperative, and epMR images in all cases (*n* = 11). The intraoperative images are T1 7-min 4-mm sequences (preresection without contrast, postresection with contrast). The epMR images are T1 sequences with contrast. Gross total resection (GTR) is defined as the absence of contrast-enhancing tissue on postoperative MRI. In this series, GTR was evaluated independently by a neuroradiologist and a pediatric neurosurgeon (EC) based on both contrast-enhanced and non-enhanced T1-weighted MR images.

### Navigation and resection control

In order to plan skin incision, craniotomy, corticotomy, and initial dissection around the tumor, in most cases (*n* = 9) we solely relied on non-contrast-enhanced intraoperative Polestar N20 images rather than high-field strength preoperative navigation images. The latter may be difficult to acquire in addition to the diagnostic scans (often made in another institution), especially in younger children requiring general anesthesia during scanning. Nevertheless, in selected cases, we relied on preoperative high-quality images as well, which are readily fused with PoleStar N20 images that are automatically imported into an integrated Stealth® computer. As such, we routinely reserved intravenous contrast for resection control images, comparing postresection non-enhanced and enhanced iMR images with preresection non-enhanced iMR and preoperative diagnostic MR images.

In five out of 11 cases, the neurosurgeon noted contrast enhancement suspect for residual tumor on initial postresection iMR images. In all five cases (including four cases of intended GTR and one case of intended subtotal resection), he continued surgery until he considered resection to be complete, as confirmed on final iMR images in four of five cases. In the other case, iMRI was nevertheless helpful in achieving a greater extent of subtotal tumor resection (case 8). Of note, in this feasibility study, any additionally resected tissue was not sent separately for histological analysis.

In one patient only (case 9), the neurosurgeon was unsure about final iMR image interpretation, revealing microcysts in the bottom of the resection cavity, adjacent to descending corticospinal tracts next to the lateral ventricle, also noted on preoperative MR images (Fig. 4). This girl had a large right postrolandic tumor (actually a DNET) with a small area of contrast enhancement in its deeper part immediately superficial to these microcysts (Fig. 4c 9a). Postresection iMR images demonstrated no residual enhancement, leaving only these microcysts that were probably of developmental origin (Fig. 4c 9d). However, the first high-field strength postoperative MRI, obtained after 3 months, did show a very small area of residual enhancement that was no longer visible on the latest MRI scan 1 year postoperatively. This girl actually was the first pediatric patient in whom we combined iMRI with intraoperative motor cortex and pyramidal tract monitoring [23] to enable maximal safe resection. Both techniques were complementary without mutual interference, given the use of platinum (non ferro-magnetic) electrodes that may be left in place even during scanning. The macroscopic, microscopic, and iMR aspect of the resection cavity as well as a slight decrease in ipsilateral pyramidal tract signal prompted us to stop resection at this point.

### Additional operation time

iMR surgery takes extra time for setup, scanning, and sometimes trouble shooting. We did not measure the amount of time for each stage in each procedure, as obviously there was also a learning curve involved. Nevertheless, with some experience, extra setup time now typically takes approximately 30 min, and preparing for an intraoperative scan takes approximately 10 min, including sterile draping of the child, returning the OR table and raising the magnet to initial scanning position, closing the StarShield®, and autocalibration of the system. Subsequent scanning time depends on the sequence and the number of sequences chosen. As previously mentioned, we almost invariably used T1 7-min 4-mm sequences before and after contrast administration and often in two of three possible planes (axial, coronal, and sagittal). Opening the StarShield®, lowering the magnet, and continuing surgery typically take no longer than 5–10 min. Therefore, the cumulative additional time for using the iMR system varies between 90 and 120 min per case, depending on the number of intraoperative scans performed.

### iMRI-related complications and limitations

We did not encounter any serious intraoperative adverse event nor any craniotomy site infection. We did encounter two cases with transient shoulder pain. Occasionally, the fragile MR-compatible cardiac electrodes or rectal thermometer were dysfunctional; however, these issues were quickly resolved by our technical staff (most often by replacing the equipment). When we first started using the PoleStar N20, we encountered interference of the magnet with the foot switch of our electrical high-speed drill (Midas-Rex Legend EHS; Medtronic, Minneapolis, MN, USA), which we solved by using a pneumatic drill instead (Midas-Rex Legend; Medtronic, Minneapolis, MN, USA). Finally, we encountered interference with the ultrasonic aspirator (CUSA Excel Ultrasonic Surgical Aspirator; Integra Radionics, Burlington, MA, USA), which we solved by moving the patients head at least 30 cm away from the magnet (i.e., moving the table upward and/or caudally) and avoiding to bring the ultrasonic aspirator to the surgical field over the magnet bore.

## Discussion

To the best of our knowledge, we are the first to report on the use of the PoleStar N20 iMR system for pediatric brain tumor resection. We present our experience regarding feasibility, intraoperative consequences, correlation between low-field strength iMRI and epMRI, as well as some limitations of this particular setup. Table 3 provides an overview of the existing literature on iMRI-guided pediatric brain tumor resection. Pathology, tumor location, and relation to eloquent areas differ between all published series. The number of cases in which the first iMR scan demonstrates residual tumor varies considerably from two of 12 (17 %) [14] to 21 of 35 (60 %) [9]. Moreover, Roth et al. [18] describe residual tumor in ten of 18 cases (56 %), including eight out of ten cases with a discrepancy between the surgeon’s judgment of the amount of tumor resected and the observed amount on subsequent iMR images.

**Table 3:** Schematic overview of the literature on iMRI-guided pediatric brain tumor resection

Author, year	Magnet strength (T)	Cases^a^	Residual tumor	Added value
Lam, 2001 [10]	1.5	7	3	1 case increased EOTR
Nimsky, 2003 [14]	0.2	12	2	2 cases increased EOTR
Samdani, 2005 [19]	0.12	15	4	4 cases increased EOTR
Kremer, 2006 [9]	0.2	35	21	only 6 cases residual tumor on epMRI
Roth, 2006 [18]	0.12	18	10	5 cases increased EOTR
Levy, 2009 [12]	1.5	49	24	24 cases increased EOTR
Present study	0.15	11	5	5 cases increased EOTR (including 4 cases with GTR)

*EOTR* extent of tumor resection, *epMRI* early postoperative MRI, *GTR* gross total resection

^a^Only those craniotomies with actual use of intraoperative MR scanning are included

### Feasibility and safety

In our experience using the PoleStar N20 mobile iMRI as an adjunct for pediatric brain tumor resection is feasible and safe. Both prone and supine positioning are rather straightforward in all age categories, especially in young children and babies whose shoulders fit in the 25 cm aperture between the magnet spools, allowing fast positioning and excellent imaging. Moreover, the smaller head of children as compared to adults allows for a larger part of the brain (in addition to the primary area of interest) to fit in the magnet’s field of view. This may be of interest for example in posterior fossa surgery, allowing near real-time observation of the resolution of a previous obstructive hydrocephalus, thus obviating the need for cerebrospinal fluid diversion. We did not encounter any serious perioperative adverse events related to using the PoleStar N20, except for transient shoulder pain in two, possibly related to downward pressure on the shoulders in a child old enough not to fit between the magnet spools with the shoulders.

Obviously, the use of iMRI encourages the surgeon to continue resection of residual tumor even close to eloquent areas such as the motor cortex or descending pyramidal tract. As near real-time intraoperative images (anatomical data) push the surgeon to maximize extent of tumor resection (EOTR) even in such delicate cases, intraoperative neuromonitoring techniques such as motor cortex and descending pyramidal tract monitoring (functional data) are of paramount importance to ensure patient safety [23]. Fortunately, using readily available platinum (non ferro-magnetic) electrodes, both techniques may be combined without any restrictions to achieve maximal safe resection even in the most daring cases.

### Intraoperative consequences of iMRI

In five out of 11 cases (45 %), an increased EOTR was achieved guided by postresection iMR images. These included four cases of intended GTR, in whom GTR was achieved after additional resection as confirmed by epMRI, and one case of intended subtotal resection, in whom additional tumor was removed and the predetermined goal of subtotal resection was achieved thanks to the information provided by postresection iMR images. In view of our protocol postponing postresection iMRI until the surgeon believed to have achieved the surgical goal (gross total or subtotal resection), our results do suggest that the use of this low-field strength iMR system enabled us to achieve an increased EOTR in five of 11 cases, including four cases in whom an intended GTR was finally achieved. Although we are well aware of many limitations when interpreting our results due to the small, retrospective, non-randomized character of the study, a prospective, randomized study examining the value of (different) iMR system(s) with regard to increasing EOTR in pediatric neuro-oncological surgery to the best of our knowledge has not been published to date.

### Correlation between iMRI and epMRI

We observed a good correlation between postresection iMR and epMR scans with regard to gross total or subtotal resection. Nevertheless, we still acquire a 1.5-T epMRI within 24 h postoperatively for resection control, planning of adjuvant treatment, and follow-up. Indeed, although PoleStar N20 MR image quality is sufficient for intraoperative decision making, a higher spatial resolution (1.5 T or more) MR scan should still be regarded as the gold standard to confirm the amount of resection.

### Limitations of the current setup

Initially, we had some technical issues with regard to the MR compatibility of patient monitoring devices (cardiac electrodes, rectal thermometer) and surgical adjuncts (electric high-speed drill, ultrasonic aspirator). These issues were resolved with help of a dedicated technical staff by replacing the electric drill with a pneumatic drill and by moving the patients head away from the magnet, respectively. To our opinion, the main issue that has not yet been solved is the unavailability of MR-compatible retractors fitting onto the headclamp during surgery. These would greatly facilitate safe removal of deep seated and skull base lesions. Ideally, such retractors should stay in place even during scanning, allowing more accurate navigation on updated intraoperative images. Unfortunately, the latter would require some major safety issues to be overcome.

## Conclusion

Using the PoleStar N20 iMR system is technically feasible and safe for both supra- and infratentorial tumor resection in children of all ages. Their rather small head and shoulders favor positioning in the magnet bore and allow the field of view to cover more than the area of primary interest, e.g., the ventricles in case of an infratentorial tumor. Standard surgical equipment may be used without significant limitations. In 45 % of our cases, using iMRI leads to an increased EOTR. Correlation between iMRI and early postoperative MRI is excellent, provided image quality is optimal and interpretation is carefully done by someone sufficiently familiar with the system. Prospective studies incorporating more children are needed to establish the precise value of iMRI in general and low-field strength iMRI in particular. Until then, early postoperative MRI remains the gold standard for planning adjuvant treatment and follow-up.
